# The relationship among college students' social support, exercise motivation, and physical exercise behavior: a cross-lagged analysis

**DOI:** 10.3389/fpubh.2026.1896136

**Published:** 2026-08-21

**Authors:** Wensi Wan, Wentao Zhang, Ting Zhang

**Affiliations:** School of Physical Education, Jiangxi Normal University, Nanchang, Jiangxi, China

**Keywords:** college students, cross-lagged analysis, exercise motivation, physical exercise behavior, social support

## Abstract

**Objective:**

This study examines the dynamic relationship among social support, exercise motivation, and physical exercise behavior, and tests the longitudinal mediating role of exercise motivation between social support and subsequent exercise behavior.

**Methods:**

Using Jiangxi Normal University as the sample institution, a three-wave cross-lagged model was employed, with measurements taken every six months (T1, T2, T3); the final matched sample comprised 576 participants. Measurements were taken using the Social Support Scale, the Exercise Motivation Scale and the Physical Activity Level Scale.

**Results:**

The findings indicate that: (1) there are significant correlations between social support, exercise motivation and physical exercise behavior, both within and across time periods; (2) Cross-lagged analysis revealed that, across every two consecutive waves, there were significant bidirectional positive longitudinal associations between social support, exercise motivation and physical exercise behavior; (3) The results of the mediation analysis showed that social support at T1 had no significant direct predictive effect on physical exercise behavior at T3 [β = 0.006, 95% CI (−0.091, 0.103), *p* = 0.903]; however, it exerted a significant positive indirect effect on physical exercise behavior at T3 via exercise motivation at T2 [β = 0.019, 95% CI (0.006, 0.032), *p* = 0.004].

**Conclusion:**

These findings suggest that external support can only lead to sustained behavioral change when it is internalized as intrinsic motivation. Consequently, interventions should prioritize stimulating and maintaining exercise motivation, whilst making full use of the mutually reinforcing feedback loop between social support and exercise motivation, in order to achieve sustainable changes in health behaviors.

## 1 Introduction

Physical exercise behavior, as one of the most critical determinants of health and an integral part of daily life, often influences mental health and forms the cornerstone of public health. Although the health benefits of physical exercise behavior are widely recognized globally—including the prevention of chronic diseases, the promotion of mental health and the enhancement of overall well-being—data from the 2022 Global Status Report on Physical Activity indicate that the age-standardized prevalence of insufficient physical exercise behavior among adults has risen significantly worldwide, with approximately 1.8 billion people globally engaging in insufficient physical exercise behavior (1). Although substantial resources have been allocated at the policy level to promote physical exercise behavior, the level of physical exercise behavior amongst university students remains unsatisfactory; these shortcomings highlight the importance of translating external support into sustained physical exercise behavior.

Social support theory and self-determination theory offer complementary perspectives on understanding how external social factors translate into sustained participation in physical exercise behavior. Social support theory explains how interpersonal relationships and social networks alleviate individual stress and promote psychological and physical well-being by providing emotional, informational and practical support (2). A wealth of research demonstrates that perceived social support from family, peers and educators is positively correlated with participation in physical exercise behavior across various population groups. However, social support alone does not guarantee behavioral persistence; its effectiveness depends to a large extent on the individual's process of psychological internalization.

Self-Determination Theory provides a robust framework for understanding this transformative process (3). At a longitudinal level, Self-Determination Theory can also explain the formation of long-term need cycles. That is to say, the basic needs satisfied through physical exercise behavior at a given point in time enhance an individual's sense of well-being and motivation to participate, which in turn promotes further physical exercise behavior at the next point in time, creating a virtuous cycle. The formation of this cycle is not determined by a single factor, but is shaped by the complex interaction between the external social environment and internal psychological processes. Among these influencing factors, social support and exercise motivation have been shown to be two key variables affecting physical exercise behavior.

## 2 Theoretical framework

### 2.1 Social support and exercise motivation

Social support refers to the assistance individuals receive from others or their environment during physical exercise behavior to achieve desired goals (2). Social support is a crucial external factor influencing the formation and change of individuals' exercise motivation. Through various forms, it can mitigate the negative effects of stress, thereby enhancing overall health and quality of life (2). The dissemination of exercise knowledge and the availability of public sports facilities are key. The attitudes and behaviors of significant others, along with professional support from teachers and instructors, significantly impact exercise motivation (4). Previous research has indicated that both parental support and peer friendship support significantly and positively predict adolescents' intrinsic motivation for exercise and their level of physical exercise behavior (5). Grounded in the core principles of self-determination theory, Wen Lu and Beihuo Hui conducted psychological assessments and constructed theoretical models among 518 college students. Their 16-week intervention study revealed that students' exercise motivation underwent a process of internalization, shifting from externally regulated behavior to intrinsic motivation under the influence of social support. These findings suggest that social support exerts a significant impact on students' autonomous motivation, exercise intention, and actual behavior, thereby reinforcing the value of social support as an effective approach for fostering the internalization of motivation and promoting sustained exercise engagement (6). In a related context, Xiao et al. conducted a cross-sectional survey of 425 social media users, integrating self-determination theory with the theory of planned behavior. Their results indicated that engagement with fitness-related social media not only directly and positively predicted exercise behavior but also strengthened intrinsic motivation by stimulating users' exercise interest, desire for challenge, and sense of competence. These findings underscore the role of intrinsic motivation as a core driving force in sustaining long-term exercise behavior (7). Social support manifests in multiple forms, including informational support, instrumental support, emotional support behaviors, and role modeling (8). In summary, Research Hypothesis 1 is proposed: Social support and exercise motivation will show a bidirectional longitudinal relationship.

### 2.2 Exercise motivation and physical exercise behavior

Motivation can be understood as an individual's desire or aversion toward a particular outcome based on different objectives, thereby altering the likelihood of achieving that outcome. Motivation can be categorized into intrinsic and extrinsic based on its source: intrinsic motivation refers to participation driven by interest or excitement derived from the activity itself, while extrinsic motivation is driven by external processes (9). Intrinsic motivation in sports originates from the internal needs of participants. The fundamental driving force behind individual engagement in physical exercise behavior lies in the fulfillment of certain human needs through such activities (10). In their study on the internal and external motivations behind physical exercise behavior among university students, Chen Shanping and colleagues pointed out that students' motivations for exercise can be divided into internal motivations (enjoyment, ability, physical appearance, health and social interaction) and external motivations (institutional requirements and compliance); stimulating internal motivations encourages university students to participate actively in physical exercise behavior, whilst overly strong external motivations may suppress students' internal motivations, making it difficult for them to maintain a long-term commitment to physical exercise behavior (11). Deci and Ryan et al. proposed Self-Determination Theory, focusing on how social environments influence human self-motivation. Its core principle is that when three innate basic psychological needs—competence, autonomy, and relatedness—are satisfied, an individual's intrinsic motivation significantly increases (3). Chu et al. found that college students' health motivation, enjoyment motivation, and competence motivation for physical exercise behavior were significantly correlated with exercise persistence, and that students of both genders recognized the health benefits of exercise (12). The subsequent effects of exercise motivation are reflected in its significant predictive role on exercise participation behavior, as exercise motivation directly influences participation levels and the degree of exercise persistence (3). Most cross-sectional studies demonstrate a correlation between exercise motivation and physical exercise behavior. Dong et al. conducted a 12-week, two-phase longitudinal questionnaire survey of 1,091 adolescents. Cross-lagged analysis revealed that intrinsic motivation unidirectionally predicted exercise behavior and subjective exercise experience 12 weeks later (13). Kwasnicka et al. posit that intrinsic motivation is a key driver for long-term maintenance of behavioral change, aligning with self-determination theory. When exercise behavior becomes internalized, exercise motivation manifests as more enduring (14). While exercise motivation influences physical exercise behavior, physical exercise behavior also progressively influences exercise motivation. In summary, Research Hypothesis 2 is proposed: exercise motivation and physical exercise behavior exhibit a bidirectional longitudinal relationship.

### 2.3 Social support, exercise motivation, and physical exercise behavior

Existing research has demonstrated links between social support, exercise motivation, and physical exercise behavior. Davison and Jago confirmed through a six-year longitudinal study that parental modeling during preadolescence and logistical support during adolescence are crucial for girls to maintain physical exercise behavior (15). Sheik et al. conducted a cross-sectional study involving 384 high school students, finding significant positive correlations between peer social support and adolescents' physical self-efficacy and physical exercise behavior. Physical self-efficacy was shown to significantly mediate the relationship between peer social support and adolescents' physical exercise behavior, revealing a unique promotional pathway for peer support in school settings (16). Social support, as a key resource in an individual's external environment, influences physical exercise behavior through direct or indirect pathways. Both social support and exercise motivation are significant positive predictors of physical exercise behavior. They influence the relationship between factors such as interpersonal relationships, body image or psychological resilience and physical exercise behavior. Promoting increased physical exercise behavior highlights the crucial role of individual and situational factors in shaping adolescent sports participation (17, 18). Li et al.'s structural equation model analysis of 2,439 U.S. students, grounded in self-determination theory, clarified that adolescents' moderate-to-vigorous physical activity (MVPA) is primarily directly influenced by intrinsic motivation, peer MVPA, and planning; Parental support indirectly influences moderate-to-vigorous physical exercise behavior through motivation (19). Social support impacts physical exercise behavior via exercise motivation and planning, serving as a core predictor of adolescent health behaviors. In summary, Research Hypothesis 3 is proposed: Exercise motivation mediates the relationship between social support and physical exercise behavior.

### 2.4 Problem statement

Based on the above literature synthesis and theoretical framework, while existing studies have separately examined the associations between social support or exercise motivation and physical exercise behavior among college students, longitudinal research examining the interplay among these three factors remains scarce. Drawing on social support theory and self-determination theory, this study posits that complex dynamic relationships may exist between social support, exercise motivation, and physical exercise behavior. The Cross-Lagged Panel Model (CLPM) is a longitudinal data analysis method used to describe the changing interrelationships or directional effects between variables over time (20). It incorporates the autoregressive effects of variables, controlling for the stability of their own sequential measurements to avoid estimation biases caused by neglecting the variables' own continuity. By comparing the magnitudes of standardized coefficients for bidirectional cross-lagged effects, it determines the direction and relative strength of longitudinal relationships between variables (21). Firstly, we acknowledge that the traditional CLPM does not adequately distinguish between stable differences between individuals and changes within individuals over time (22). However, the research question in this study is essentially at the inter-individual level: it aims to examine whether individuals who report higher levels of social support subsequently demonstrate stronger exercise motivation and engage more actively in physical exercise behavior. Traditional CLPM is able to address this issue directly and concisely (23). Furthermore, given the three-wave design with a six-month interval between waves, traditional CLPM is sufficiently statistically powerful and converges more stably than RI-CLPM. Given this, this study employs a cross-lagged panel model to explore the dynamic relationships among the variables.

## 3 Methods

### 3.1 Participants

This study employed a three-wave longitudinal design, with a six-month interval between each wave. Participants were recruited from Jiangxi Normal University in Nanchang, Jiangxi Province, China, using cluster random sampling. Twenty-two classes were randomly selected from among all first-year undergraduate students in non-sports-related programmes at the university. All students in these 22 classes were invited to participate in the study on a voluntary basis. The first survey was conducted in December 2024, with a total of 635 questionnaires collected. After excluding invalid data (including repetitive responses, widespread errors and omissions, etc.), 541 valid questionnaires were retained, representing a valid response rate of 85.2%. The second survey was conducted in June 2025, with 585 participants completing the questionnaire; 531 valid questionnaires were retained, yielding a valid response rate of 90.8%. The third survey was conducted in December 2025, with 605 participants completing the questionnaire; 508 valid questionnaires were retained, yielding a valid response rate of 84.0%. To ensure data quality and make full use of the available longitudinal data, two inclusion criteria were applied: (a) participants must have completed at least two of the three survey rounds; (b) their coding must have remained consistent and traceable across all survey rounds. Following the exclusion of participants who did not meet these criteria, the final analysis sample comprised 576 participants, of whom 57 were missing at T1, 70 at T2 and 94 at T3. The mean age of the participants was 18.30 ± 0.66 years; the distribution of demographic variables is shown in Table 1.

**Table 1:** Participant demographic information (*N* = 576).

Variable	Category	*N*	%
Gender	Male	356	61.8
	Female	220	38.2
Origin	Rural	282	49.0
	Urban	294	51.0
Academic discipline	Science and engineering	285	49.5
	Humanities, history and philosophy	103	17.9
	Economics, management, education and law	140	24.3
	Agriculture, medicine and military science	1	0.2
	Art and physical education	47	8.2

An attrition analysis was subsequently conducted, using ‘completion of at least two survey rounds' as the grouping criterion, to compare demographic variables and study variables between dropout and non-dropout participants. The chi-squared test revealed no significant difference in gender distribution (χ^2^ = 0.173, *p* = 0.68). An independent-samples t-test was performed using T1 data; the results showed no significant differences between the two groups in terms of social support (t = 0.541, *p* = 0.59), exercise motivation (t = −0.177, *p* = 0.86) and physical exercise behavior (*t* = −1.61, *p* = 0.11). At the same time, Little's test was performed on the data; if *p* < 0.001, the data were not completely at random (MCAR) (24). The results showed that there was no significant difference between the pattern of missing data and completely random missing data (χ^2^ = 356.418, df = 347, *p* = 0.352), indicating that the outcome variable conforms to the completely random missing data assumption. Consequently, missing values in the variables were handled using the Full Information Maximum Likelihood (FIML) estimation method in Mplus 8.0 software.

### 3.2 Procedure

During the preparatory phase, contact was made with the homeroom teacher of the target class to proactively explain the purpose of the visit and research objectives. After obtaining the teacher's consent, the target class and testing time were confirmed through active communication with both the homeroom teacher and the physical education instructor. To ensure smooth administration, the questionnaire was completed during physical education class. Before distribution, the examiner explained the content and requirements to students, emphasizing that there were no right or wrong answers and clarifying confidentiality principles to gain their trust. Paper-and-pencil tests were used, with questionnaires distributed and collected on-site. The administration procedures were largely consistent across all three sessions. This study has been reviewed and approved by the Ethics Review Committee of the Physical Education College, Jiangxi Normal University.

### 3.3 Measures

#### 3.3.1 Social support scale

The Social Support Scale adapted by Duan Mengshuang was employed, measuring four dimensions: family support, friend support, informational support, and instrumental support. Each item was rated on a 5-point Likert scale ranging from 1 (strongly disagree) to 5 (strongly agree), with higher scores indicating greater perceived social support (25). In this study, the Cronbach alpha coefficients for the scale were 0.918 (T1), 0.933 (T2), and 0.930 (T3), respectively. Confirmatory factor analysis (CFA) results demonstrated good construct validity for the social support scale across three time points, with S-B χ^2^/df = 2.65–3.63, CFI = 0.921–0.942, TLI = 0.900–0.931, RMSEA = 0.054–0.071, SRMR = 0.040–0.058.

#### 3.3.2 Simplified version of the exercise motivation scale (MPAM-R)

This study employed the Chinese version of the Exercise Motivation Scale, simplified and compiled by Chen Shanping et al. The scale comprises 15 items organized into five theoretical dimensions: health motivation, appearance motivation, enjoyment motivation, competence motivation, and social motivation. Each item is rated on a 5-point Likert scale ranging from 1 (“not at all true”) to 5 (“very true”), with higher scores reflecting greater motivation (26). In this study, the Cronbach alpha coefficients for the scale were 0.950 (T1), 0.951 (T2), and 0.951 (T3), respectively. Confirmatory factor analysis results demonstrated good construct validity for the Exercise Motivation Scale across three time points, with S-B χ^2^/df = 2.91–4.01, CFI = 0.927–0.939, TLI = 0.905–0.920, RMSEA = 0.063–0.077, SRMR = 0.036–0.043.

#### 3.3.3 Physical activity rating scale (PARS-3)

The Physical Activity Rating Scale (PARS-3), revised by Liang Deqing et al., was used to assess physical activity levels across three dimensions: exercise intensity, frequency, and duration. Each dimension was scored on a 1–5 scale, with the formula: Physical Activity Level = Exercise Intensity × (Exercise Duration – 1) × Exercise Frequency. Physical activity levels were categorized as follows: ≤ 19 points = low activity; 20–42 points = moderate activity; ≥43 points = high activity. The higher the score, the more physical exercise behavior university students engage in. The test-retest reliability of this questionnaire was 0.821 (27). In the study, the total scores calculated using the formula were analyzed at three time points (T1, T2, T3) as individual indicators of physical exercise behavior.

### 3.4 Data analysis

Statistical analysis was performed using SPSS 26.0 to conduct descriptive statistics, correlation analysis, independent samples t-tests, chi-squared tests for dropout analysis, Little's MCAR test, and Harman's one-factor test for common method bias on the longitudinal data from the three waves of matching and aggregation, in order to assess the current status of social support, exercise motivation and physical exercise behavior. Model estimation was carried out using Mplus 8.0 software, employing robust maximum likelihood estimation (MLR) (28). Prior to model specification, confirmatory factor analysis and longitudinal measurement invariance testing were conducted to establish the psychometric adequacy of the constructs over time. Subsequently, a cross-lagged model was established; to simplify the model and enhance statistical power, four nested models were sequentially tested and compared (29). Model 1 (M1) served as the baseline model with all path coefficients freely estimated. Building upon M1, the following models were specified: M2 constrained autoregressive path coefficients to be equal across time while freely estimating cross-lagged path coefficients; M3 constrained cross-lagged path coefficients to be equal across time while freely estimating autoregressive path coefficients; M4 constrained both autoregressive and cross-lagged path coefficients to be equal across time. Calculate the chi-squared values and degrees of freedom differences for the four models. If ΔS-B χ^2^ does not reach significance under Δdf (*p* > 0.05), it indicates no significant model deterioration, and the model with constrained path coefficients should be selected (30). Model fit was evaluated using a comprehensive set of fit indices, including the Satorra–Bentler scaled chi-square to degrees of freedom ratio (S-B χ^2^/df), Comparative Fit Index (CFI), Tucker-Lewis Index (TLI), Root Mean Square Error of Approximation (RMSEA), and Standardized Root Mean Square Residual (SRMR). Acceptable model fit was determined based on established criteria: S-B χ^2^/df ≤ 5, CFI and TLI ≥0.90, and RMSEA and SRMR ≤ 0.08 (31). The analysis of the mediating effect was based on a three-variable cross-lagged model. Using bias-corrected Bootstrap estimation with a 95% confidence interval, gender—a key demographic variable—was included in the model as a covariate to control for the baseline effects of such variables, thereby enhancing the reliability of the test results for the mediating effect.

In the cross-lagged model, social support and exercise motivation were treated as latent variables, whilst physical exercise behavior was included as an observed variable, as it was calculated as a single total score using the standard PARS-3 formula. To improve the model fit, the two variables—social support and exercise motivation—were clustered based on the mean scores of their respective theoretical dimension subscales (32). Specifically, the social support scale, comprising 17 items, was clustered into four clusters based on family support, friend support, informational support and instrumental support; the exercise motivation scale, comprising 15 items, was clustered into five clusters based on health motivation, appearance motivation, enjoyment motivation, competence motivation and social motivation.

## 4 Results

### 4.1 Common method bias test

As the data for this study were collected via a questionnaire and all originated from the same participants, there may be a risk of common method bias. In terms of questionnaire design, the physical activity rating scale, social support scale and exercise motivation scale were organized into separate sections, with unrelated distractor items inserted to break the participants' continuity of thought between consecutive items; data collection was conducted on a fully anonymous basis, with participants completing the questionnaire independently. The questionnaire instructions explicitly stated that there were no right or wrong answers. This served to minimize the likelihood of common method bias.

During the data analysis stage, a Harman one-factor test was performed on the data. The first measurement revealed six factors with eigenvalues greater than 1, with the first factor explaining 34.714% of the variance. The second measurement revealed six factors with eigenvalues exceeding 1, with the first factor explaining 35.053% of the variance. The third measurement showed six factors with eigenvalues above 1, and the first factor accounted for 37.216% of the variance. All values were below the critical threshold of 40% (33). However, some scholars have pointed out that the Harman one-factor test has limitations; therefore, confirmatory factor analysis was conducted on all items using Mplus 8.0; The results indicated that the single-factor model fitted the data extremely poorly: S-B χ^2^/df = 7.06–7.99, CFI = 0.539–0.559, TLI = 0.510–0.532, RMSEA = 0.112–0.117, SRMR = 0.135–0.156 (34). These results indicate that there is no serious common-factor bias in this study.

### 4.2 Descriptive statistics and correlation analysis

The correlation matrix of variables across different time points is shown in Table 2. The results indicate that the autocorrelation coefficients for social support, exercise motivation, and physical exercise behavior are all significant and range from moderate to high, suggesting that the variables exhibit sufficient stability over time. Significant correlations exist among the variables at the same time point, and significant correlations also exist between variables at different time points, indicating a relatively stable relationship among the three variables. Therefore, the results satisfy the conditions of cross-temporal stability and synchronous correlation, meeting the assumptions of the cross-lagged model. The demographic variable of gender (coded as 1 = male, 2 = female) was significantly correlated with the core variables; it was included as a covariate in subsequent analyses to reduce confounding bias.

**Table 2:** Descriptive statistics and correlations among study variables (*N* = 576).

Variable	M ±SD	1	2	3	4	5	6	7	8	9	10
1. Gender	1.38 ± 0.49	1									
2. Social support (T1)	3.25 ± 0.68	0.001	1								
3. Social support (T2)	3.37 ± 0.71	−0.114^*^	0.284^**^	1							
4. Social support (T3)	3.33 ± 0.65	−0.088	0.248^**^	0.342^**^	1						
5. Exercise motivation (T1)	3.47 ± 0.84	−0.090^*^	0.416^**^	0.189^**^	0.212^**^	1					
6. Exercise motivation (T2)	3.46 ± 0.82	−0.209^**^	0.281^**^	0.367^**^	0.296^**^	0.517^**^	1				
7. Exercise motivation (T3)	3.38 ± 0.79	−0.151^**^	0.131^**^	0.417^**^	0.421^**^	0.435^**^	0.570^**^	1			
8. Physical exercise behavior (T1)	16.11 ± 15.52	−0.211^**^	0.323^**^	0.205^**^	0.250^**^	0.380^**^	0.289^**^	0.238^**^	1		
9. Physical exercise behavior (T2)	19.31 ± 17.06	−0.253^**^	0.228^**^	0.239^**^	0.227^**^	0.289^**^	0.349^**^	0.345^**^	0.473^**^	1	
10. Physical exercise behavior (T3)	19.58 ± 16.81	−0.215^**^	0.169^**^	0.289^**^	0.308^**^	0.254^**^	0.392^**^	0.451^**^	0.443^**^	0.551^**^	1

Gender was coded as 1 = male, 2 = female. M = mean, SD = standard deviation. ^**^p < 0.01, ^*^p < 0.05.

### 4.3 Measurement invariance

Prior to constructing the cross-lagged model, confirmatory factor analysis was applied to the measurement instruments. Configural invariance, metric invariance, and scalar invariance were sequentially tested for the Social Support Scale and Exercise Motivation Scale used in this study. In the test results, if the change in ΔCFI was less than 0.01 and the change in ΔRMSEA was less than 0.015, it indicated invariance across different time points (35). At minimum, configural invariance and metric invariance constitute the foundational conditions for constructing cross-lagged models (36). However, when comparing latent variable means, scalar invariance is required (37).

Table 3 presents the results of the longitudinal invariance tests for the social support and exercise motivation models. The results indicate that the social support model satisfies configural, metric, and scalar invariance, and the exercise motivation model also satisfies configural, metric, and scalar invariance. Based on the model fit indices and the nested model comparisons, both measurement instruments demonstrated adequate longitudinal measurement invariance, supporting their suitability for the subsequent cross-lagged panel analysis.

**Table 3:** Longitudinal measurement invariance.

MODEL	S-B *χ^2^*	df	CFI	TLI	SRMR	RMSEA (90% CI)	ΔCFI	ΔRMSEA
Social support								
M1 Configural invariance	144.069	38	0.956	0.923	0.040	0.070 [0.058, 0.082]		
M2 metric invariance	163.566	44	0.950	0.925	0.056	0.069 [0.058, 0.080]	0.006	0.001
M3 scalar invariance	179.658	50	0.946	0.928	0.059	0.067 [0.057, 0.078]	0.004	0.002
Exercise motivation								
M1 Configural invariance	163.934	72	0.982	0.973	0.026	0.047 [0.038, 0.057]		
M2 metric invariance	185.844	80	0.979	0.972	0.038	0.048 [0.039, 0.057]	0.003	0.001
M3 scalar invariance	197.673	88	0.978	0.974	0.039	0.047 [0.038, 0.055]	0.001	0.001

### 4.4 Cross-lagged analysis

Table 4 presents the fit indices and model comparison results of the cross-lagged model of social support, exercise motivation, and physical exercise behavior with gender as a covariate. The results show that, compared with M1, M2 and M3, the fit of M4—which was subject to the strictest equivalence constraints—did not deteriorate significantly; consequently, M4 was selected as the more concise model.

**Table 4:** Fit indices of cross-lagged models and model comparison.

MODEL	Fit Indices						Model comparison			
	S-B χ^2^	df	CFI	TLI	RMSEA	SRMR	Model	ΔS-B χ^2^	Δdf	*p*
M1	1,146.488	395	0.921	0.907	0.058	0.045				
M2	1,145.889	398	0.921	0.908	0.057	0.046	M2 vs. M1	−0.484	3	1
M3	1,158.401	401	0.920	0.907	0.057	0.049	M3 vs. M1	10.350	6	0.111
M4	1,159.304	404	0.920	0.908	0.057	0.050	M4 vs. M1	11.241	9	0.263
							M4 vs. M2	12.288	6	0.056
							M4 vs. M3	0.807	3	0.848

**Figure 1:**
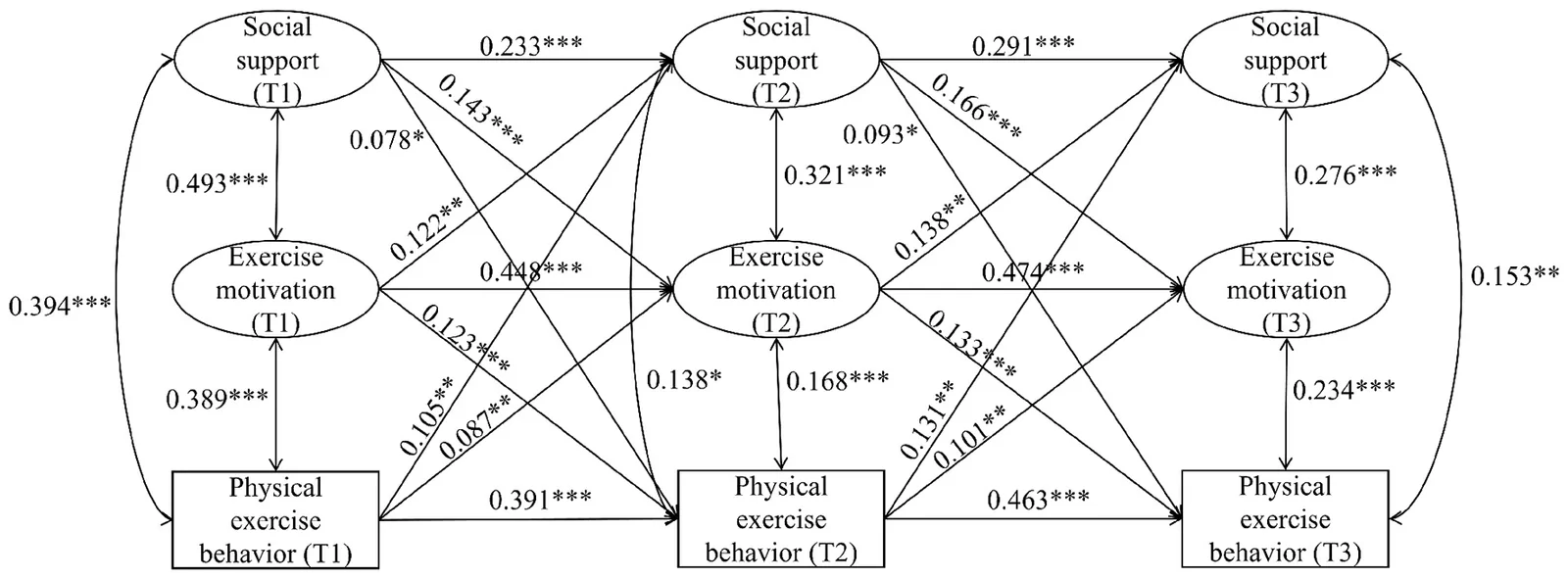
Cross-lagged panel model of social support, exercise motivation, and physical exercise behavior. Gender was included in the analysis as a covariate, but to ensure the clarity of the figures, these pathways and non-significant paths have been omitted; ***p < 0.001, **p < 0.01, *p < 0.05.

The cross-lagged model of social support, exercise motivation, and physical exercise behavior is shown in Figure 1. The model fit indices, with S-B χ^2^/df = 2.87, CFI = 0.920, TLI = 0.908, RMSEA = 0.057, SRMR = 0.050 were acceptable. In the autoregressive path analysis, all three variables—social support, exercise motivation, and physical exercise behavior—exhibited good cross-temporal stability, with all autoregressive paths reaching statistical significance (*p* < 0.001).

Model standardized path coefficients are presented in Table 5. The cross-lagged model indicates that there are longitudinal correlations between the variables. Social support at T1 significantly and positively predicted exercise motivation at T2 (β = 0.143, *p* < 0.001), whilst social support at T2 significantly and positively predicted exercise motivation at T3 (β = 0.166, *p* < 0.001); Conversely, exercise motivation at T1 also positively predicted social support at T2 (β = 0.122, *p* = 0.001), and exercise motivation at T2 positively predicted social support at T3 (β = 0.138, *p* = 0.002). Exercise motivation at T1 was a positive predictor of physical exercise behavior at T2 (β = 0.123, *p* < 0.001), and exercise motivation at T2 was a positive predictor of physical exercise behavior at T3 (β = 0.133, *p* < 0.001); Physical exercise behavior at T1 predicted exercise motivation at T2 (β = 0.087, *p* = 0.005), whilst physical exercise behavior at T2 predicted exercise motivation at T3 (β = 0.101, *p* = 0.006). Social support at T1 directly predicted physical exercise behavior at T2 (β = 0.078, *p* = 0.036), and social support at T2 directly predicted physical exercise behavior at T3 (β = 0.093, *p* = 0.039), It is worth noting that social support at T1, across the two measurement waves, did not significantly predict physical exercise behavior at T3 (β = 0.006, *p* = 0.903), indicating that social support at T1 does not directly influence physical exercise behavior at T3; physical exercise behavior at T1 significantly predicted social support at T2 (β = 0.105, *p* = 0.007), and physical exercise behavior at T2 significantly predicted social support at T3 (β = 0.131, *p* = 0.01).

**Table 5:** Standardized path coefficients.

Path type	Specific path	β	SE	*p*	95% CI
Autoregressive paths	T1SS → T2SS	0.233	0.054	0.000	[0.128, 0.339]
	T2SS → T3SS	0.291	0.066	0.000	[0.161, 0.420]
	T1EM → T2EM	0.448	0.040	0.000	[0.370, 0.526]
	T2EM → T3EM	0.474	0.044	0.000	[0.388, 0.560]
	T1PEB → T2PEB	0.391	0.045	0.000	[0.304, 0.478]
	T2PEB → T3PEB	0.463	0.052	0.000	[0.361, 0.565]
Cross-lagged paths	T1SS → T2EM	0.143	0.036	0.000	[0.072, 0.213]
	T2SS → T3EM	0.166	0.046	0.000	[0.077, 0.256]
	T1SS → T2PEB	0.078	0.037	0.036	[0.005, 0.151]
	T2SS → T3PEB	0.093	0.045	0.039	[0.005, 0.181]
	T1EM → T2SS	0.122	0.038	0.001	[0.048, 0.195]
	T2EM → T3SS	0.138	0.045	0.002	[0.049, 0.226]
	T1EM → T2PEB	0.123	0.033	0.000	[0.058, 0.188]
	T2EM → T3PEB	0.133	0.038	0.000	[0.059, 0.206]
	T1PEB → T2SS	0.105	0.039	0.007	[0.028, 0.182]
	T2PEB → T3SS	0.131	0.051	0.010	[0.031, 0.230]
	T1PEB → T2EM	0.087	0.031	0.005	[0.026, 0.148]
	T2PEB → T3EM	0.101	0.037	0.006	[0.029, 0.173]
	T1SS → T3PEB	0.006	0.050	0.903	[−0.091, 0.103]

SS, social support; EM, exercise motivation; PEB, physical exercise behavior.

In summary, there is a bidirectional positive predictive relationship between social support and exercise motivation, with the two factors reinforcing one another. Exercise motivation is a key factor in promoting physical exercise behavior, whilst social support can also have a direct impact on physical exercise behavior in the short term.

### 4.5 Mediation effect analysis

This study employed a CLPM to estimate longitudinal mediating effects. Building upon a three-variable cross-lagged model, gender was specified as a covariate, and the test was conducted using the bootstrap method with 5,000 repeated samples (38). The results indicated that exercise motivation plays a significant and complete mediating role between social support and physical exercise behavior. Specifically, the indirect effect of T1 social support on T3 physical exercise behavior via T2 exercise motivation was significant [β = 0.019, SE = 0.007, 95% CI (0.006, 0.032), *P* = 0.004], whilst the direct effect of T1 social support on T3 physical exercise behavior was not significant [β = 0.006, 95% CI (−0.091, 0.103), *p* = 0.903].

## 5 Discussion

### 5.1 Correlation analysis of social support, exercise motivation, and physical exercise behavior

The results of the descriptive statistics show that, from T1 to T3, exercise motivation exhibited a slight but sustained downward trend, which may reflect the cumulative impact of academic pressure and time constraints during the first year of university. Furthermore, across the three waves of data, scores for physical exercise behavior consistently remained below the “low activity” threshold, with considerable individual variation; this indicates that a significant proportion of the sample lacks sufficient physical exercise behavior, and that there are marked differences between individuals in terms of exercise participation. Thirdly, the stability of exercise motivation between adjacent waves was slightly higher than that of social support and physical exercise behavior. This is consistent with the view that motivation, as a relatively stable psychological disposition, exhibits greater temporal persistence than situational or behavioral variables (39, 40). The correlations provide preliminary support for the theoretical framework. At all three time points, social support and exercise motivation mostly exhibited moderate positive correlations, which is consistent with the findings of previous cross-sectional studies (41). he correlations between social support and physical exercise behavior were both significant, but the T1 to T3 correlation was relatively low; this result suggests that the long-term direct effect of social support on physical exercise behavior may be limited, and that its influence is likely mediated primarily through psychological processes—a premise consistent with the indirect pathway hypothesis proposed by Self-Determination Theory (42). Importantly, the significant correlations between pairs of variables across consecutive waves provide empirical support for estimating a complete lag model. Furthermore, the relatively weak correlation between social support and exercise behavior further reinforces the validity of testing the mediating role of exercise motivation. In summary, these findings not only support previous correlational evidence but also reveal that social support may influence long-term physical exercise behavior through the internalization of motivation.

### 5.2 Cross-lagged analysis of social support, exercise motivation, and physical exercise behavior

The results of the cross-lagged model indicate that there is a bidirectional lagged relationship between social support and exercise motivation; a finding consistent with prior research and supporting Hypothesis 1 (43). Specifically, individuals who perceive greater social support tend to report stronger exercise motivation. Self-determination theory posits that the value of physical exercise behavior lies not solely in external rewards and evaluations, but primarily in the psychological fulfillment gained through autonomy, competence, and social connectedness (44). As an external environmental factor, social support can stimulate and reinforce exercise motivation by fulfilling individuals' needs for belonging and competence. Exercise motivation, as an internal driver, prompts individuals to actively seek, perceive, and maintain social support, forming a bidirectional cycle. This aligns closely with the core tenets of self-determination theory proposed by Ryan & Deci et al. (3). Exercise motivation at an earlier time point can positively predict physical exercise behavior at a later time point, and physical exercise behavior at an earlier time point can also positively predict exercise motivation at a later time point. These findings are consistent with previous research (13), and Hypothesis 2 is supported. Autonomous motivation serves as an endogenous force enhancing adolescents' exercise persistence (45). Even with ample external support, individuals lacking intrinsic motivation remain unlikely to engage in physical exercise behavior. Physical exercise behavior is a significant predictor of exercise motivation, which is consistent with some studies suggesting that physical exercise behavior itself may automatically translate into stronger exercise motivation. Furthermore, research has found that social support is a significant predictor of physical exercise behavior in the short term (46), the influence of social support is not limited to short-term, direct effects, but may also involve long-term mechanisms mediated through other variables. Physical exercise behavior can also exert a reciprocal influence on exercise motivation and social support; it is precisely the motivation, enthusiasm and social initiative generated by the behavior itself that truly activate support networks (44). This finding suggests that social support and exercise motivation are longitudinally associated with physical exercise behavior.

### 5.3 Longitudinal mediating effects of social support, exercise motivation, and physical exercise behavior

The key finding of this study is that exercise motivation mediates the longitudinal relationship between social support and physical exercise behavior. T1 social support → T2 exercise motivation → T3 physical exercise behavior (indirect effect β = 0.019, *p* = 0.004; direct effect β = 0.006, *p* = 0.903). The indirect effect via exercise motivation was significant, whereas the direct effect of social support on subsequent physical exercise behavior was not significant. These results indicate that exercise motivation fully mediates the relationship between social support and physical exercise behavior over time, supporting Hypothesis 3. Previous research has shown that incorporating government and institutional support into the social support framework not only helps university students master exercise methods, but also subtly fosters positive exercise motivation through institutional emphasis, thereby promoting students' cognitive understanding and ability to adopt a healthy lifestyle (47). Our findings further support this view by clarifying the temporal sequence: social support does not directly drive long-term behavioral change; its influence is transmitted entirely through the enhancement of exercise motivation at an intermediate stage. This suggests that the process of motivation internalization does not occur solely in the present moment, but unfolds gradually, requiring a period of consolidation before it translates into sustained behavioral outcomes. Social support indirectly promotes physical exercise behavior by enhancing students' exercise motivation, a finding also validated by Wu et al. (48). From a theoretical perspective, this model of complete mediation is consistent with the core principles of SDT. SDT posits that a social environment which supports autonomy, a sense of competence and a sense of belonging contributes to the internalization of extrinsic motivation, thereby promoting more autonomous forms of behavioral regulation. The findings reveal that motivation acts as a full mediator between support and behavior, suggesting that the quality of motivation is a proximate determinant of sustained behavior, rather than merely the presence of support provided by others. According to social support theory, social support primarily promotes an individual's physical and mental health by buffering the adverse effects of stress (1). This buffering perspective suggests that social support may influence health behaviors through both direct pathways (e.g. stress reduction) and indirect psychological mechanisms (e.g. motivation). The findings suggest that, in the context of long-term physical exercise behavior, the indirect route via motivation is the primary mechanism, whilst the influence of the direct route is negligible once motivational factors are taken into account. This does not negate the buffering role of social support, but indicates that its impact on behavior is primarily realized through motivational processes.

Finally, the findings of this study provide practical guidance for university mental health practitioners to promote healthy behaviors among students. Intervention strategies should not be limited to simply recommending physical exercise behavior to students who lack the habit of exercising in a one-way manner. Instead, practitioners should recognize that students with weak social support or insufficient motivation to exercise may be caught in a self-perpetuating cycle: a lack of supportive relationships undermines their willingness to exercise, which in turn leads to further avoidance of physical exercise behavior and a missed opportunity to improve their health. Rather than directly encouraging students to engage in physical exercise behavior, practitioners can design group counseling sessions or peer support programmes to first enhance students' perceived social support. Given that social support at adjacent stages plays a significant predictive role in exercise motivation, early intervention to strengthen social support systems (such as family, friends and informational support) can lay a solid foundation for boosting students' motivation to engage in physical exercise behavior; this approach is more sustainable than short-term behavior-oriented interventions. Given that exercise motivation plays a crucial mediating role between social support and physical exercise behavior, university mental health counselors can collaborate with campus sports departments to jointly design integrated mind-body health programmes. For example, referral mechanisms could be established to direct students identified during counseling as having low exercise motivation to sports instructors for exercise guidance aimed at boosting their motivation, whilst counselors continue to provide ongoing social support. It is important to explicitly guide students to internalize the reasons for exercising as intrinsic motivation, as this process of internalization serves as the psychological bridge linking social support to sustained physical exercise behavior. These recommendations are highly aligned with the “Healthy China 2030” initiative, which emphasizes the integration of mental health promotion and physical exercise behavior into the university environment (49). This enhances the practical effectiveness of public mental health services, helps foster long-term exercise habits among university students, and improves their overall physical and mental well-being.

## 6 Limitations and future directions

Although this study employed a cross-lagged model to elucidate the dynamic relationships among social support, exercise motivation, and physical exercise behavior—offering advantages over cross-sectional studies in revealing temporal relationships—it retains certain limitations that warrant refinement and expansion in subsequent research.

Firstly, the data were collected from a single university. Although heterogeneity across disciplines was achieved by sampling 22 classes, the findings may not be generalisable to students in other regions, institutions or cultural contexts. Future research should seek to validate these findings using multi-site and cross-cultural samples. Secondly, all data in this study were derived from self-report scales completed by university students regarding social support, exercise motivation and physical exercise behavior. Although these scales have been widely used and have demonstrated good reliability in previous research, self-reported data are susceptible to recall bias, social desirability bias and subjective misinterpretation. Future research should incorporate objective data from multiple sources. Thirdly, we employed a traditional CLPM rather than a RI-CLPM. Whilst the CLPM allows for the examination of between-individual differences, it cannot fully distinguish between between- and within-individual variance. The RI-CLPM offers an alternative approach that distinguishes stable differences in personality traits from within-individual fluctuations. As our primary research interest lies in longitudinal associations between individuals, and given that the CLPM is widely accepted in this field, we opted for the traditional CLPM. Nevertheless, future studies with larger sample sizes and more measurement time points could employ latent variable growth curve models or random-intercept bidirectional lag models to gain a more nuanced understanding of the internal dynamics of individuals over time. Finally, regarding sample attrition, the analysis showed no significant differences between retained participants and those who dropped out in terms of gender, level of social support at T1, exercise motivation or physical exercise behavior. Little's MCAR test further confirmed that the pattern of missing data was consistent with completely random missing data. These results suggest that attrition bias is unlikely to have had a significant impact on the study findings. Nevertheless, it remains necessary to conduct future studies with larger sample sizes and more frequent follow-ups to further minimize the potential impact of attrition.

## 7 Conclusion

This study employed a three-wave cross-lagged model to reveal the dynamic interrelationships between social support, exercise motivation and physical exercise behavior across two six-month time periods. The findings confirmed the existence of a dynamic, bidirectional positive relationship between social support, exercise motivation and physical exercise behavior over a six-month period, and further validated the role of exercise motivation—as a key mediating variable—in linking social support to subsequent physical exercise behavior. Specifically, the direct effect of social support at T1 on physical exercise behavior at T3 was not significant (β = 0.006, *p* = 0.903); however, social support indirectly promoted subsequent exercise behavior by enhancing exercise motivation at the intermediate stage (specific indirect effect = 0.019, *p* = 0.004). These findings suggest that external support can only lead to sustained behavioral change when it is internalized as intrinsic motivation. Consequently, interventions should prioritize stimulating and maintaining exercise motivation, whilst making full use of the mutually reinforcing feedback loop between social support and exercise motivation, in order to achieve sustainable changes in health behaviors.

## Data Availability

The original contributions presented in the study are included in the article/supplementary material, further inquiries can be directed to the corresponding author.
